# Cross-Layer Federated Learning for Lightweight IoT Intrusion Detection Systems

**DOI:** 10.3390/s23167038

**Published:** 2023-08-09

**Authors:** Suzan Hajj, Joseph Azar, Jacques Bou Abdo, Jacques Demerjian, Christophe Guyeux, Abdallah Makhoul, Dominique Ginhac

**Affiliations:** 1 Imagerie et Vision Artificielle (ImVIA) Laboratory, Université de Bourgogne Franche-Comté, 21078 Dijon, France; 2 Femto-St Institute, UMR 6174 CNRS, Université de Franche-Comté, 25030 Besançon, France; 3 School of Information Technology, University of Cincinnati, Cincinnati, OH 45221, USA; 4 LaRRIS, Faculty of Sciences, Lebanese University, Fanar P.O. Box 90656, Lebanon; 5 Computer Science & IT Department, Faculty of Arts and Sciences, Holy Spirit University of Kaslik (USEK), Jounieh P.O. Box 446, Lebanon

**Keywords:** federated learning, internet of things, lightweight intrusion detection, lightweight sampling, semi-supervised learning

## Abstract

With the proliferation of IoT devices, ensuring the security and privacy of these devices and their associated data has become a critical challenge. In this paper, we propose a federated sampling and lightweight intrusion-detection system for IoT networks that use K-meansfor sampling network traffic and identifying anomalies in a semi-supervised way. The system is designed to preserve data privacy by performing local clustering on each device and sharing only summary statistics with a central aggregator. The proposed system is particularly suitable for resource-constrained IoT devices such as sensors with limited computational and storage capabilities. We evaluate the system’s performance using the publicly available NSL-KDD dataset. Our experiments and simulations demonstrate the effectiveness and efficiency of the proposed intrusion-detection system, highlighting the trade-offs between precision and recall when sharing statistics between workers and the coordinator. Notably, our experiments show that the proposed federated IDS can increase the true-positive rate up to 10% when the workers and the coordinator collaborate.

## 1. Introduction

Intrusion detection is a critical component of security systems for Internet of Things (IoT) security. The proliferation of connected devices and the increasing amount of data being transmitted create opportunities for malicious actors to exploit vulnerabilities [1]. An essential challenge in developing effective intrusion-detection systems for IoT applications is handling large volumes of data while preserving data privacy and minimizing energy consumption [2]. Network nodes experience diverse traffic patterns, causing standalone intrusion-detection system (IDS) nodes to learn only from accessible traffic. This leads to delays in attack detection and potential privacy breaches if collaborative IDS are used, as sensitive information may be shared across nodes. To address this challenge, we propose a lightweight federated sampling and intrusion-detection approach based on an adaptation of the K-means clustering algorithm called the Baseline K-means algorithm.

The Baseline K-means algorithm is a semi-supervised novelty detection technique that trains a classifier using a limited amount of labeled benign data and subsequently employs it to label additional unlabeled data points. This method is particularly advantageous when unlabeled data are abundant but a scarcity of benign data. The proposed model utilizes two centroids, one for the baseline distribution and another for the anomalous distribution, and employs the Mahalanobis distance as a distance metric to account for the distribution’s covariance. Compared to standard K-means clustering that uses Euclidean distance, this approach allows for more robust and accurate identification of anomalous behavior.

The primary goal of this paper is to propose a federated, lightweight, and privacy-preserving intrusion-detection system for lightweight IoT environments. We present a novel approach for real-time intrusion detection in IoT applications by combining a federated version of the Baseline K-means algorithm with a cluster-based sampling method, referred to as the “cross-layer” federated IDS. The proposed approach tackles the challenge at two layers, the first being sampling which significantly reduces the memory, power, and computational resources needed to process with overwhelming traffic. The second layer is anomaly detection which copes with the reduced traffic by learning from other nodes, through a coordinator, in a federated learning process. The cross-layer approach allowed us to shift clustering into the sampling layer and thus co-optimizing the performance of both layers. The proposed solution is a cross-layer with a significant reduction in memory, power, and computational resources and an increase in detection capabilities without impacting individual nodes’ privacy.

The cluster-based sampling technique, proposed in our previous work [3], ensures a high level of data representation, including rare subgroups, effectively reducing the sampling error due to data variance. This technique is a prerequisite for our current work, as it serves as the foundation for our proposed federated Baseline K-means algorithm. The cluster-based sampling algorithm initially applies a feature reduction to the available packets to decrease the overhead, based on a study highlighting the essential features for detecting various attacks in the NSL-KDD dataset [4]. The algorithm has multiple advantages as [3] showed, the first being significant efficiency, compared to other sampling algorithms, for very low sampling rates which resembles a lightweight IoT environment. The second is longer traffic visibility which is important against slow-rate attacks [5,6,7]. Subsequently, the K-means clustering algorithm is applied to divide the data stream into separate homogeneous groups, called clusters. The algorithm then samples these clusters proportionally to a chosen sampling rate, ensuring that the overall representation of the cluster data remains consistent even after sampling.

In this approach, multiple IoT devices passively participate in the training process by computing their local statistics, such as means and distances to the benign distribution, which are then transmitted to a central coordinator for aggregation. This allows each IoT node’s model to be updated with recent and more representative statistics without transmitting sensitive data, effectively preserving data privacy. Moreover, by employing cluster-based sampling before the intrusion-detection process, we reduce the processing energy consumption and the volume of data to be transmitted, therefore extending the lifetime of the IoT node. The federated Baseline K-means algorithm offers a practical, scalable, and privacy-preserving solution for intrusion detection in IoT applications while also addressing energy efficiency concerns. In this paper, we provide a comprehensive explanation of our proposed federated Baseline K-means algorithm and its integration with the cluster-based sampling technique, as well as experimental results demonstrating the effectiveness and efficiency of our approach for intrusion detection in IoT applications.

The motivation for this work stems from several challenges faced by lightweight IoT nodes. First, their limited resources make running IDS difficult, yet neglecting this security measure leaves them vulnerable. Second, collaborative IDS systems risk privacy breaches by exchanging sensitive traffic information. Lastly, these nodes may function in remote or hostile conditions where information exchanged between collaborative IDSs can be both intentionally disrupted and naturally corrupted.

This work makes several key contributions. First, it develops a cross-layer federated learning system tailored for lightweight IoT nodes, utilizing cluster-based sampling to reduce memory usage and employing lightweight IDS to minimize computational requirements while ensuring high detection rates. Second, it ensures privacy by sharing only the model’s statistics with the aggregator, thus preserving collaborative learning without exposing sensitive information. Lastly, by limiting the exchanged information to these statistics, the work reduces both the risk of adversarial jamming and noise-induced errors, while also enabling local IDS to function independently if completely isolated from other nodes. The experiments and obtained results validate the objective of this paper, showing that the merging and sharing of statistics between the coordinator and the workers enhance the performance of the IoT nodes over time, increasing their capabilities to detect intrusions more accurately.

This paper is organized as follows: Section 2 reviews the related work, discussing lightweight IDS and sampling algorithms for IoT, federated learning for IoT IDS, and the cluster-based sampling algorithm proposed in our previous work, which is employed in the cross-layer IDS of the present study. Section 3 provides an in-depth description of the proposed work, beginning with an explanation of the term “cross-layer” in the context of our research. The proposed semi-supervised novelty detection algorithm, based on the K-means algorithm and referred to as Baseline K-means, is then introduced, followed by a discussion of the federated version of this technique. Section 4 outlines the experiments conducted in this paper, interprets the results, and highlights how the proposed approach achieves the study’s objectives. Section 5 offers a comprehensive analysis of the observations related to the experiments and their outcomes, addresses the limitations of the proposed work, and suggests future research directions. Finally, Section 6 concludes the paper.

## 2. Related Work

### 2.1. Lightweight IDS for IoT

Cyber security has become increasingly challenging due to the proliferation of the Internet of Things (IoT), where a massive number of tiny, smart devices push trillion bytes of data to the Internet and is expected to reach 73.1 ZB (zettabytes) by 2025 [8]. IoT devices have limited computational capabilities and thus researchers have shifted their focus onto designing lightweight IDS that can deliver the needed security requirements while operating on those thin devices.

Zarpelão et al. [9] surveyed IDS developments for IoT and discovered a growing interest in lightweight IDS. The authors discovered two tracks that claim to be lightweight which are:

- Signature-based lightweight IDS (such as [10]): this track is beyond the scope of this work.
- Anomaly-based lightweight IDS: We will focus this work on this research track.

Lee et al. [11] detected 6LowPAN attacks by observing IoT nodes’ reported energy consumption. To deal with energy consumption attacks, Le et al. [12] created a lightweight intrusion-detection system that restricts sensing operations to cluster heads, allowing the remaining nodes to operate normally. This approach is aligned with Reza et al. [13]. Jan et al. [14] concentrated on creating computationally lightweight IDS using support vector machines, supervised machine learning (ML), which does not limit the IDS to a single attack type (as in [11]) nor to the number of nodes running the IDS (such as in [12,13]).

By limiting the number of investigated features, Soe et al. [15] developed a lightweight anomaly-based IDS strategy that selects the features with the highest gain ratio and discards all others, thus reducing the amount of computation required. It is worth noting that this strategy runs the risk of missing out on rare attacks that can only be detected using discarded features. This method is consistent with that proposed by Davahli et al. [16], where feature selection is based on the hybridization of a genetic algorithm (GA) and the Grey Wolf Optimizer (GWO).

Khater et al. [17] combined the last two strategies (feature reduction and supervised deep learning) to enhance the communication security of lightweight IoT devices in a Fog computing environment. To maintain the lightweight criteria, a combination of Modified Vector Space Representation (MVSR) N-gram (1-gram and 2-gram) were used for system call encoding in the feature extraction phase while using a sparse matrix for space reduction. Then, the extracted features were fed into a Multilayer Perceptron (MLP) model with a single hidden layer that would classify the nature of the network traffic.

Instead of being selective on the features (such as in [15]) or on nodes (such as in [12,13]), Sedjelmaci et al. [18] proposed a strategy that is selective on time. The authors proposed a game-theoretic approach for identifying the times when the attacks are most probably going to happen. Only then, the IDS functionality is enabled.

Deep Neural Networks (DNN) were applied in the hope of improving the detection accuracy of lightweight IDS. One of the most recent applications is “Realguard” by Nguyen et al. [19], a DNN-based IDS that implements a simple MLP with 5 hidden layers. Realguard can run on low-end IoT devices while achieving high attack detection accuracy.

### 2.2. Sampling Algorithms for IDS

IoT devices cannot handle all sent data due to rising network overhead and stagnant power storage capacity. Researchers have turned to sampling methods before data analysis to mitigate this, reducing data volume. This approach must prevent information loss to avoid compromising threat detection accuracy. Sampling techniques are designed to optimize IDS efficiency and attack detection accuracy. IoT nodes sample packets, creating a subset of network traffic for subsequent analysis and detection. The success of a sampling method depends heavily on factors such as the sampling rate and the chosen strategy.

A network-based IDS (NIDS) analyzes data samples as network packets. Thus, the population is all packets in our network traffic, whereas the subset is a selection. Since only a specific number of packets are taken for analysis, the essential parameter is the sampling rate, or sampling ratio, which determines the ultimate size of the subset compared to the original population. Some sampling algorithms may produce an incorrect sample size. Static and dynamic sampling algorithms exist. A static sampling process is conducted periodically or randomly following a given rule or data interval. We can classify those rules under three main categories of sampling decisions: count-based, time-based, and content-based. Every static algorithm that samples data based on its ordering position in a stream of packets is identified as count-based. A time-based algorithm focused on the arrival time of a packet (timestamp). Finally, content-based sampling methods analyze the content of the packet before data selection. As this final method increases the overhead and computation time, content-based algorithms, known as well as filtering algorithms, are beyond the scope of our research. The main advantage of using a static sampling algorithm would be reducing bandwidth and storage requirements, as only a subset is detained for anomaly detection analysis. In their turn, dynamic or adaptive sampling algorithms use different sampling intervals and/or rules for data sample decisions.

In this context, several studies have looked at the effects of data sampling. Mai et al. [20] investigated, using various sampling algorithms, the effect of sampling high-speed IP-backbone network traffic on intrusion-detection outcomes, specifically port scans and volume anomaly detection. Roudiere et al. [21] tested the accuracy of the “Autonomous Algorithm for Traffic Anomaly Characterization” detector in detecting DDoS attacks over sampled traffic. Various sampling policies were used to sample the traffic. The authors of [22,23] investigated how packet sampling influenced anomaly detection results. Silva et al. [24] proposed a framework for evaluating packet sampling’s effects. They examined the effectiveness of each sampling algorithm and proposed a set of metrics for assessing each sampling technique’s ability to produce a representative sample of the original traffic. Bartos et al. [25] investigated the impact of traffic sampling on anomaly identification and presented a new adaptive flow-level sampling algorithm to improve the sampling process’ accuracy. Using traces containing the Blaster worm, Brauckhoff et al. [26] assessed the accuracy of existing anomaly detection and data sampling algorithms. Liu et al. [27] implemented a novel Difficult Set Sampling Technique (DSSTE) to tackle the class imbalance problem which helped in the detection of rare attacks. They used Edited Nearest Neighbor (ENN) to identify the difficult set then applied the K-means algorithm to compress the majority in the difficult set, and finally augmented the data of the clusters to obtain the final sample. A more thorough discussion can be seen in our previous survey [28] and benchmarking [4] works where we investigated all data sampling strategies, their impact on detecting various attacks, and the behavior and robustness of features under various sampling strategies. We also looked at how the estimation of network features varies depending on the sampling method, sample size, and other factors, and how this affects statistical inference from these data.

### 2.3. Federated Learning for IoT IDS

In the evolving landscape of IoT applications, ensuring data privacy has become a paramount concern. With increasing connectivity and data sharing among devices, there is a growing need for robust strategies to safeguard sensitive information. One such strategy is enhancing the ‘opacity’ of a system, a concept discussed extensively in the realm of cyber-physical systems (CPSs). This method involves creating a system that is inscrutable to external observers, thereby protecting its ‘secret’ state information [29]. Apart from enhancing opacity, another well-known machine-learning approach for preserving privacy is federated learning. Federated learning is a distributed machine-learning technique that allows multiple IoT nodes to collaboratively train a global model without sharing their raw data with a centralized server. In the context of intrusion detection, federated learning can be used to detect attacks on IoT devices without compromising the privacy of individual devices. In this direction, Pei et al. [30] designed a sophisticated data aggregation method that allows network traffic anomaly detection based on the self-coding of long- and short-term memory networks without violating privacy. Attota et al. [31] followed another approach to ensuring privacy. They used multi-view classification and multi-view ensemble learning to deliver better prediction accuracy without breaching privacy. In the same direction, Mothukuri et al. [32] designed a federated learning process using Gated Recurrent Units. All the previous methods are vulnerable to data poisoning attacks, such as backdoor-attacks, as Nguyen et al. showed [33]. To deal with data poisoning attacks, Generative Adversarial Networks are used with federated learning such as in [34,35].

Saadat et al. [36] studied the performance of hierarchical federated learning compared to federated learning in an IoT context and showed that despite its infrastructure overhead, hierarchical federated learning proved its superiority over federated learning in training loss, testing accuracy, and convergence speed. This work is aligned with Sarhan et al. [37] where a hierarchical federated learning framework has been designed to operate using blockchain. Federated learning in IoT IDS has been extensively studied and benchmarked in the literature such as in [38,39,40,41,42,43].

In the framework of this study, a federated intrusion-detection system plays a vital role in safeguarding data privacy. By enabling each IoT device to execute local anomaly detection and retain its raw data, while only transmitting summary statistics to a central aggregator, the system enhances its anomaly detection capabilities and significantly reduces the transfer of sensitive information. This approach adeptly addresses the dual necessities of effective anomaly detection and robust privacy protection within the context of resource-limited IoT settings.

## 3. Lightweight Semi-Supervised Intrusion Detection

K-means clustering is a feasible solution for IoT intrusion detection due to recent advancements in IoT devices such as Arduino and ESP32. It is a lightweight and efficient algorithm that can be run on small devices depending on data size and computational resources. Its computation and memory requirements depend on the number of data points, features, and clusters, making it suitable for small datasets and a small number of clusters.

This section presents the proposed Baseline K-means for intrusion detection in IoT and a federated implementation of the same approach.

### 3.1. Cross-Layer

As indicated in Section 2.1 and Section 2.2, sampling and intrusion detection have been studied separately in the context of lightweight IoT. This separation creates abstract layering which is typical in communication stacks and provides significant advantages such as modularity. In this work, we are interested in investigating the feasibility of a cross-layer design incorporating sampling and intrusion detection and ultimately federated learning. To the best of our knowledge, this work is the first to join sampling and intrusion detection in the context of lightweight IoT.

The literature contains cross-layer designs for IoT IDS, but the term cross-layer is used to indicate different meanings. Amouri et al. [44] proposed a cross-layer IoT IDS that spans MAC and Network layers. In the same direction, Canbalaban and Sen [45] proposed a cross-layer IoT IDS that spans link and routing layers. Long et al. [46] proposed cross-layer industrial IoT IDS that spans its three layers which are: the application layer, the network layer, and the perception layer. This work is aligned with Malik et al. [47] and Kore and Patil [48]. A more thorough analysis was conducted by Parween et al. [49], but none of the surveyed works incorporated sampling and intrusion detection.

### 3.2. Baseline K-Means

Novelty detection is a class of semi-supervised learning techniques that involves training a classifier on a small amount of labeled data (in this case, the benign class) and then using it to label additional unlabeled data points. The newly labeled data points can then be added to the training set to improve the classifier’s performance. This approach can be useful when there is a large amount of unlabeled data available, but only a small amount of labeled benign data.

The proposed lightweight approach is an adaptation of the K-means technique, known as the Baseline K-means. It is a semi-supervised novelty detection approach that uses a small amount of labeled data, considers it as the baseline for learning, and trains a classifier to label additional unlabeled data points. The proposed approach involves training a model on a set of training data and using it to evaluate new observations. Outliers are defined as observations that differ significantly from the distribution of the training data, even if they form a high-density region. To adapt the K-means algorithm for intrusion detection, the approach of using a distance measure to a known baseline was adopted. Specifically, the Mahalanobis distance between each data point and the mean of the normal (benign) data were used as the distance metrics. The Mahalanobis distance accounts for the covariance of the distribution and provides a measure of the distance between a point and a distribution. The Mahalanobis distance *d* from a point *x* to a distribution with mean μ and covariance matrix Σ is given by:

$$D_{M}\left(x,\mu ,\Sigma \right)=\sqrt{{\left(x-\mu \right)}^{⊤}\Sigma^{-1}\left(x-\mu \right)}$$

where *T* denotes the transpose of a matrix.

The proposed model uses two centroids: one for the baseline distribution and one for the anomalous distribution. During training, data points are assigned to the nearest centroid based on Mahalanobis distance. If a point is closer to the baseline centroid, it is classified as the baseline. If it is farther from the baseline centroid than a certain threshold times the distance to the anomalous centroid, it is classified as anomalous and added to the anomalous cluster. When there are enough anomalous points, the anomalous centroid is updated as the farthest point from the baseline distribution. The proposed process is illustrated in Algorithm 1 and repeated for a certain number of iterations or until convergence. The update() function in this algorithm updates the baseline and anomalous statistics (mean, covariance, and centroid) based on the current state of the data. It computes Mahalanobis distance from each baseline point to the baseline centroid to determine the threshold for classifying new points as baseline or anomalous. It also computes the mean and covariance of the anomalous data and sets the anomalous centroid. The centroids array is updated to reflect the new baseline and anomalous centroids.
**Algorithm 1:** Baseline K-means fit function. 
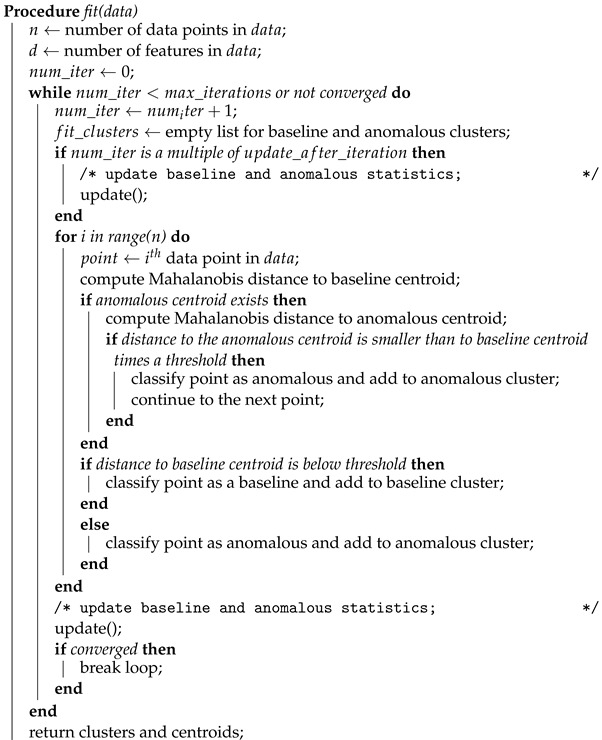


During prediction, the model computes the Mahalanobis distances from each data point to the baseline distribution and the anomalous centroid. It classifies each data point as baseline or anomalous based on the same distance threshold as during training. It then assigns each data point to the nearest centroid and returns the assigned cluster as 0 for benign and 1 for anomalous. This process is illustrated in Algorithm 2.

A high-level overview of the Baseline K-means class is presented in Algorithm 3. This class is specifically designed for intrusion detection using K-means clustering. It is constructed with four input arguments: baseline data, maximum number of iterations, percentile, and update after iteration values:

- baseline_data: an array representing the baseline (benign) data to be used for training the model.
- max_iterations: an optional integer argument that specifies the maximum number of iterations to be performed during training.
- percentile: an optional integer argument that specifies the percentile to use for computing the threshold distance between baseline and anomalous data points.
- update_after_iteration: an optional integer argument that specifies the number of iterations after which to update the baseline and anomalous statistics.

**Algorithm 2:** Baseline K-means predict function. 
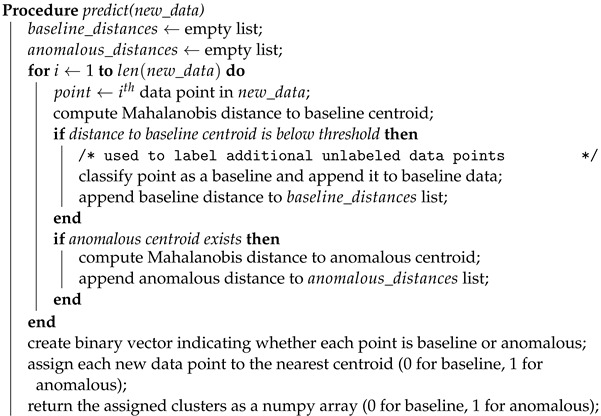


**Figure 1:**
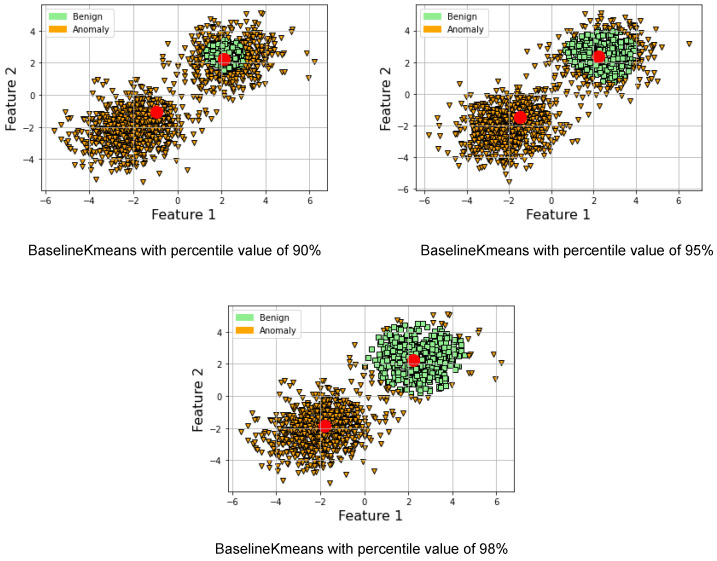
These plots illustrate the clustering of data based on different percentile values (90, 95, and 98) used to compute the anomaly threshold. Red markings represent the centroids of each cluster. As the percentile value rises, more data points located at the extremities of the baseline centroid are categorized as benign, resulting in fewer benign data points being labeled as anomalous. On the other hand, a reduction in the percentile value results in a higher number of benign data points being identified as anomalous.

Please note that the importance of the percentile argument in this class is to compute the threshold distance that separates the baseline data from the anomalous data. Specifically, it sets the percentile of the Mahalanobis distances between each baseline data point and the baseline centroid that should be used as the threshold. By setting the percentile to 90, for example, the threshold will be set such that 90% of the distances between each baseline data point and the baseline centroid are below the threshold, and 10% of the distances are above it. This helps to distinguish the baseline data from the anomalous data, which are likely to be farther away from the baseline centroid. Figure 1 illustrates how the percentile value affects the clustering of points around the benign centroid. Reducing the percentile value used to calculate the threshold in the Baseline K-means class will result in a lower threshold, which means that more data points will be classified as anomalous. This is because the threshold is calculated based on the Mahalanobis distance from the baseline centroid to the data points. A lower percentile means that a smaller proportion of the data points will be used to calculate the threshold, resulting in a lower threshold that is more likely to include data points that are further from the baseline centroid. As a result, more benign data points will be classified as anomalous because their distance from the baseline centroid is greater than the lower threshold. Therefore, a lower percentile value will lead to a higher false-positive rate and more benign data points being labeled as anomalous. It is worth noting that the computational complexity of our method is primarily influenced by factors such as the number of features and data points but not necessarily detection accuracy. The detection accuracy, on the other hand, is determined largely by the quality of the data and the selected detection threshold. As to how we determine the detection threshold, it is currently set based on our experience, including trial-and-error experimentation and considerations specific to the dataset we are using. The goal has been to select a threshold that maximizes recall.
**Algorithm 3:** Baseline K-means class shown in a Python-like pseudocode. **Input**: Baseline data *B*, Anomalous data *A*, max iterations *I*, percentile *P*, update after iteration *U* **Output**: Trained model with updated centroids and threshold **Class Baseline K-Means:** 
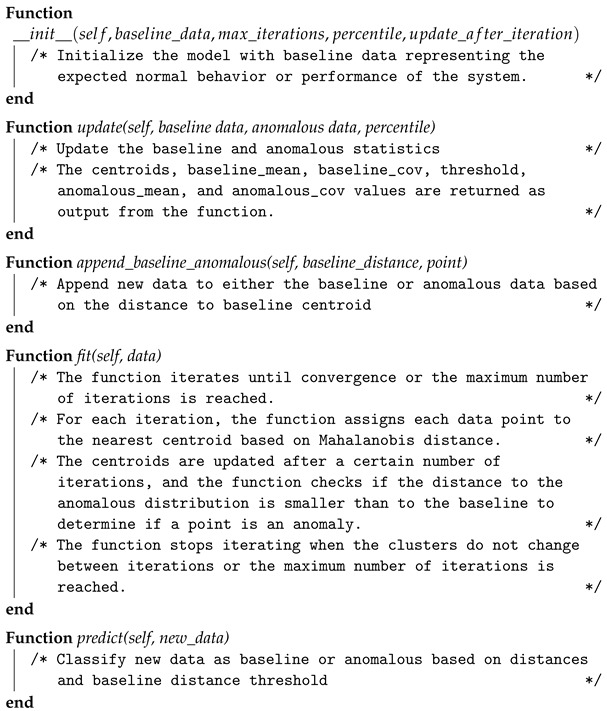


This approach is specifically designed for intrusion detection, where the baseline distribution represents the normal behavior of a system and the anomalous distribution represents the behavior of potential attackers. The ability to set a threshold allows for tuning the sensitivity of the detector, balancing between false positives and false negatives while prioritizing the true-positive rate. The use of the Mahalanobis distance and covariance matrix enables the algorithm to take correlations between features into account and adjust the distance metric to the specific distribution of the data. In contrast to standard K-means, which uses the Euclidean distance to compute the distance between data points and the centroid, this implementation uses the Mahalanobis distance. As a result, this approach can handle data points with different variances and covariances. It should be noted that the cluster centroids are not updated during the K-means algorithm as is carried out in the standard K-means algorithm. Instead, the baseline and anomalous centroids are set based on the mean of their respective data sets. Overall, this implementation provides a more robust and accurate approach to identifying anomalous behavior.

### 3.3. Federated Sampling and Intrusion Detection

**Figure 2:**
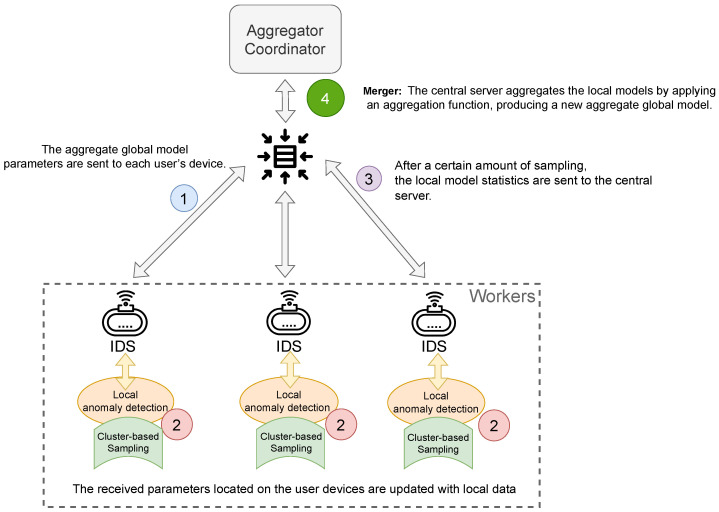
The figure outlines a federated intrusion-detection strategy for IoT networks. The BaselineKMeans class, acting as coordinator, initiates baseline data and fits the model, sending statistics to the workers. Workers implement a cluster-based sampling algorithm, processed by the WorkerKmeans IDS, and assign labels to the data points using coordinator statistics. Workers then send their statistics for coordinator updates, merging worker data to refresh the global model. This method diminishes latency, data transmission, and privacy issues typical of centralized intrusion-detection systems.

This section presents a proposed lightweight federated sampling and intrusion-detection approach. The approach is based on the federated Baseline K-means algorithm, which is a distributed and privacy-preserving version of the Baseline K-means algorithm designed for intrusion detection in IoT applications. The federated version of the algorithm can train a clustering model without transmitting sensitive data, therefore preserving data privacy. In the federated version, multiple IoT devices participate in the training process and compute their own local statistics, including means and distances. The statistics are then transmitted to a central coordinator for aggregation. The coordinator updates the baseline and anomalous centroids based on the merged statistics from the IoT devices and computes a new threshold. It is worth noting that the local intrusion-detection step occurs after the cluster-based sampling to reduce energy consumption related to processing and transmission. The federated Baseline K-means algorithm offers a practical and scalable solution for real-time intrusion detection in IoT applications while preserving data privacy. Figure 2 illustrates the proposed approach.

As shown in Figure 2, each individual IDS node calculates the statistics of the local model and shares it with the aggregator which in turn collects all the statistics from all the IDS nodes. Then, the aggregator updates its model and returns the global model parameters to the IDS nodes. This is performed in a star network topology and peer-to-peer application architecture. The aggregator, in addition to being an IDS node, is selected to execute the aggregator role as an additional task. The aggregator role can rotate on the participating IDS nodes, similar to [50].

The algorithm involves four key steps as illustrated in Figure 2: Initialization, Local clustering, Share cluster statistics, and Merge statistics. In the Initialization step, the coordinator initializes the K-means clustering model with a fixed number of clusters and shares cluster statistics between workers (Algorithm 4). Each node uses the global representation of the benign cluster and the distance threshold to detect anomalies in its local data subset. Data points with distances above the threshold are classified as anomalies, while data points with distances below the threshold are classified as benign. In the Local clustering step, each node uses its own local K-means clustering model on its own sampled data. Each node calculates the Mahalanobis distance to the benign centroid for its individual local data points. During the cluster statistics sharing step, every node shares statistics like cluster centroids and distances to the benign centroid with other nodes via the coordinator, but not the data itself. Please note that the workers do not perform fitting since the clustering model is already initialized by the coordinator. This approach centralizes the processing burden on the coordinator, enabling a lightweight implementation for the IoT workers/nodes. Finally, in the Merge statistics step (Algorithm 5), the coordinator merges the cluster statistics from each node to create a global representation of the benign and anomalous clusters. This can be done by averaging the cluster centroids and defining a classification threshold for the Mahalanobis distances based on the global representation of the benign cluster. The updated global model can then be shared with the worker nodes.
**Algorithm 4:** The coordinator’s export statistics function is presented in Python-like pseudocode. It utilizes the pseudoinverse (pinv) with regularization to ensure the covariance matrix’s invertibility and numerical stability **Output**: Threshold, baseline mean, inverse baseline covariance, anomalous mean, inverse anomalous covariance **Export statistics function:** 
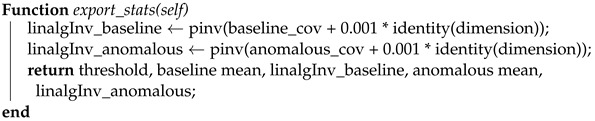


**Algorithm 5:** The merge statistics function of the coordinator, shown in a Python-like pseudocode **Input**: Worker baseline means, worker anomalous means, distances from baseline points to new baseline centroid, distances from anomalous points to anomalous centroid **Output**: Updated threshold and centroids **Merge statistics function:** 
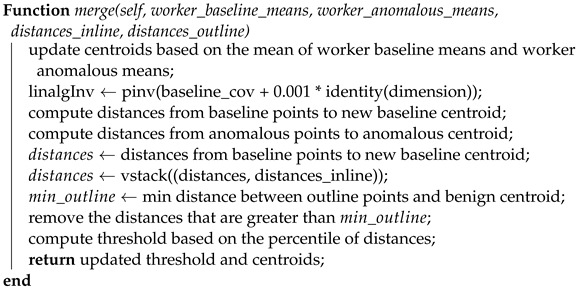


## 4. Experimental Setup and Simulations

In this section, we provide an overview of the experiments and simulations conducted in this study to validate the relevance of the proposed solution. Initially, we introduce the dataset utilized in this research and justify our rationale for employing a semi-supervised novelty detection approach. Subsequently, we compare the proposed lightweight IDS based on K-means clustering with other semi-supervised novelty detection methods. The results elucidate the reasons behind selecting this particular solution for IoT intrusion detection in comparison with existing alternatives. Furthermore, we present the simulations conducted using the federated IDS, demonstrating that sharing and merging statistics between workers and the coordinator effectively enhance the performance of the IoT IDS. Finally, we discuss the simulations pertaining to the cross-layer IDS, wherein intrusion detection is applied following the cluster-based sampling, and examine the impact of sampling rate on IDS performance. The Baseline K-means has been implemented with Python. The main used libraries are scikit-learn, pandas, and numpy. We publicly shared the Python code on GitHub for reproducibility [51].

### 4.1. NSL-KDD Dataset and Semi-Supervised Learning

The discussed techniques are implemented using Python and tested against the NSL-KDD dataset [52]. The NSL-KDD dataset is a widely used and improved version of the original KDD Cup 1999 dataset [53], specifically designed for evaluating intrusion-detection systems (IDS). It contains a diverse set of network traffic data, including both normal (benign) and various types of malicious (intrusion) instances. The NSL-KDD dataset addresses some of the inherent problems of the original KDD dataset, such as redundant records and data imbalance, making it a more suitable choice for evaluating the performance of intrusion-detection techniques.

In the context of IoT intrusion detection, adopting a semi-supervised novelty detection approach using K-means presents numerous advantages over traditional supervised and unsupervised learning methods. Supervised learning approaches are hindered by the need for labeling network traffic, which can be time-consuming and resource-intensive, particularly in dynamic IoT environments. Conversely, unsupervised learning approaches are only effective when the data contains clearly separable clusters of samples, making it challenging to accurately identify and classify network attacks.

**Figure 3:**
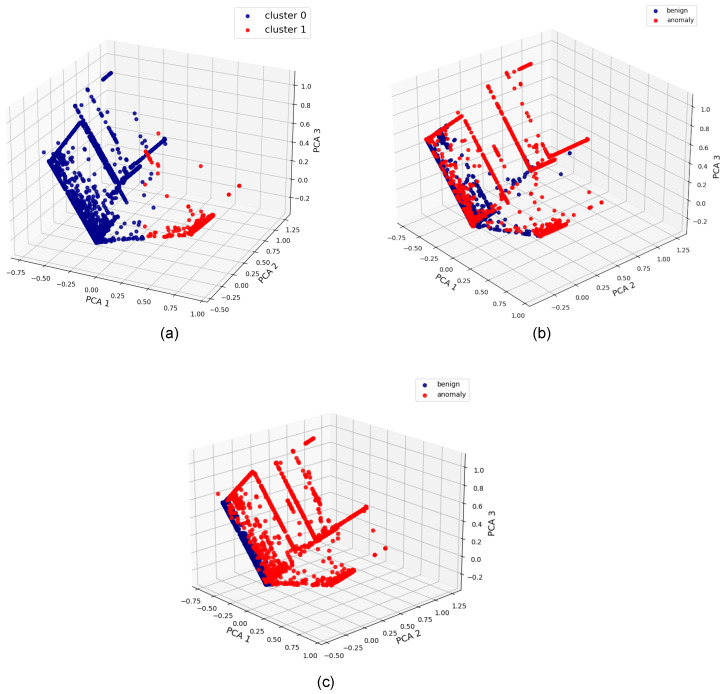
Clustering the NSL-KDD data points using the standard unsupervised K-means and the proposed semi-supervised Baseline K-means with k = 2. (**a**) Scatter plot of 10,000 data points with the unsupervised K-means predicted classes (transformed with PCA). (**b**) Scatter plot of 10,000 data points with ground truth labels (transformed with PCA). (**c**) Scatter plot of 10,000 data points with the semi-supervised Baseline K-means (percentile = 95) predicted classes (transformed with PCA).

When applying the standard K-means algorithm to network traffic datasets such as NSL-KDD, it faces certain challenges. To better illustrate the motivation for using semi-supervised learning, we visualized the NSL-KDD data transformed with principal component analysis (PCA) and compared the results of unsupervised K-means clustering, ground truth, and the proposed Baseline K-means. As shown in Figure 3a, the K-means clustering has difficulty grouping clusters of “anomalous” labels and often misclassifies a significant portion of benign traffic as attack traffic. Although adjusting the value of k can help to a certain degree, the visual analysis suggests that there may be fundamental issues with relying solely on unsupervised clustering methods to classify network attacks on these data.

Figure 3b (ground truth) reveals a region where benign and anomalous data are difficult to separate. By adopting a semi-supervised novelty detection approach using K-means (Figure 3c), we aim to address these limitations and enhance the effectiveness of IoT intrusion-detection systems. Semi-supervised novelty detection leverages the strengths of both supervised and unsupervised learning techniques to address these issues. This approach enables intrusion detection even when labeled data are scarce, which is often the case in IoT environments. By incorporating prior knowledge of normal traffic behavior, semi-supervised novelty detection can more effectively distinguish between benign and malicious activities in the network. Moreover, this method is more adaptive to the evolving nature of cyber threats, as it can identify novel attacks without requiring explicit knowledge of their characteristics. However, this approach comes with a trade-off, as seen in Figure 3c. Many benign data points are classified as anomalous due to the nature of the proposed technique that prioritizes the true-positive rate over the false-positive rate.

### 4.2. Semi-Supervised Novelty Detection for Intrusion Detection

This section compares our proposed Baseline K-means algorithm with several other prominent novelty detection techniques. These novelty detection techniques share a commonality with our proposed approach, as they all initially utilize a portion of the benign data to learn the underlying patterns of normal traffic. This aspect is crucial for effectively identifying and classifying anomalous traffic in IoT environments, where labeled data are often scarce. Furthermore, these algorithms can be conveniently implemented using the widely used scikit-learn library, a popular open-source machine-learning library in Python.

By comparing our proposed Baseline K-means algorithm with the below alternative novelty detection techniques, we aim to demonstrate the effectiveness and suitability of our approach for IoT intrusion-detection tasks:

- One-Class Support Vector Machine (SVM): One-Class SVM is a popular machine-learning algorithm that constructs a decision boundary around the benign data points in the feature space. It aims to maximize the margin between the benign samples and the origin, allowing for the detection of novel data points that do not conform to the learned benign pattern.
- One-Class Gaussian Mixture Model (GMM): One-Class GMM is a generative probabilistic model that assumes the benign data are generated from a mixture of several Gaussian distributions. It estimates the parameters of these Gaussian distributions using the Expectation-Maximization (EM) algorithm. During the classification phase, it computes the likelihood of a new data point belonging to the learned benign distributions, identifying anomalies based on a predefined threshold.
- Local Outlier Factor (LOF): LOF is a density-based algorithm that detects outliers by comparing the local density of a data point with the densities of its neighbors. The algorithm assigns an outlier score to each data point based on the ratio of its local density to the average density of its nearest neighbors. Points with significantly lower densities than their neighbors are considered anomalies.
- Isolation Forest: Isolation Forest is an ensemble-based anomaly detection algorithm that builds multiple decision trees. Each tree isolates data points by randomly selecting features and splitting them based on random thresholds. The isolation of an anomalous data point requires fewer splits compared to benign data points, resulting in a shorter path length. The algorithm calculates an anomaly score based on the average path length across all trees in the ensemble.
- Minimum Covariance Determinant (MCD): MCD is a robust statistical method for detecting outliers based on the Mahalanobis distance, which takes into account the correlation between features. MCD estimates the mean and covariance matrix of the benign data by finding the subset of data points with the smallest covariance determinant. It then computes the Mahalanobis distance between each data point and the estimated mean, identifying anomalies that exceed a predefined threshold.

In our simulation, we utilized the NSL-KDD dataset, which contains approximately 120,000 rows of data, with each row representing a feature vector containing multiple features. To evaluate the performance of each semi-supervised novelty detection algorithm, we initially presented each method with 500 benign data points to learn the patterns of normal traffic. Subsequently, for each sliding window of 1000 data points, the novelty detection techniques were applied to labelize and classify the data. Precision, recall, and F1-score were calculated and recorded for each method within each window. After processing the entire dataset, we computed the average values for precision, recall, and F1-score across all windows for each novelty detection technique, enabling a comprehensive comparison of their performance in the context of IoT intrusion detection.

**Figure 4:**
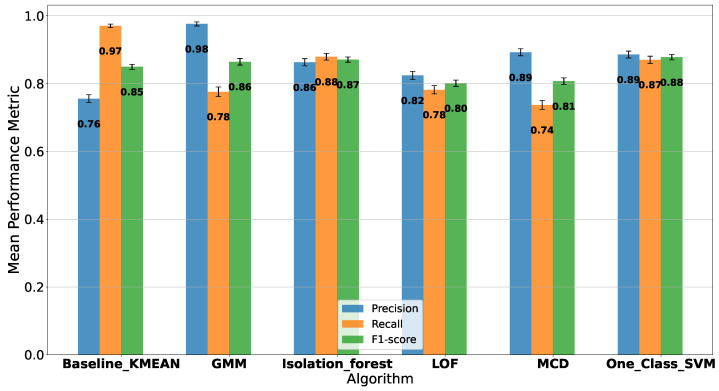
Performance comparison of semi-supervised novelty detection algorithms for intrusion detection: The bar plot illustrates the precision, recall, and F1-score for each algorithm, highlighting the proposed Baseline K-means’ ability to prioritize the true-positive rate (highest recall) while maintaining a competitive F1-score, making it suitable for security and IDS applications.

The results of the simulation for each semi-supervised novelty detection algorithm are presented in Figure 4. Upon interpreting the results, it is evident that the proposed Baseline K-means algorithm achieves the highest recall at 0.97, indicating its ability to prioritize the true-positive rate. This is particularly important for security and intrusion-detection system (IDS) applications, where the primary focus is detecting as many intrusions as possible. Although the precision of the proposed method is lower compared to other algorithms, the F1-score, which is a harmonic mean of precision and recall, remains competitive at 0.85. The One-Class SVM has the highest F1-score at 0.88, but its recall is lower than the proposed Baseline K-means. This suggests that although the One-Class SVM has a balanced performance in terms of precision and recall, it may not prioritize the true-positive rate as much as the proposed approach. Considering the nature of security and IDS applications, the proposed Baseline K-means algorithm is a suitable choice due to its strong emphasis on maximizing the true-positive rate, ensuring a robust detection of intrusions in the network.

### 4.3. Federated Lightweight IDS

In this section, we describe the simulation of a federated intrusion-detection system (IDS) consisting of one coordinator and three workers. The simulation starts with the coordinator learning a baseline model from an initial dataset containing N (in this case, N = 100) benign data points. After the initial training, the coordinator exports its statistics to the three workers. In the proposed version of the federated IDS, the coordinator actively participates in the processing of network traffic data alongside the three workers. Unlike the traditional federated learning setup where the coordinator only serves to aggregate and distribute updates, in this case, the coordinator is also responsible for analyzing new data that it has not encountered during the initial training phase.

The NSL-KDD dataset contains approximately 120,000 rows of data, which translates to around 30 windows or epochs for our simulation. During each epoch, the coordinator and the three workers process 1000 data points, amounting to a total of 4000 data points per epoch. This design choice allows us to evaluate the performance and effectiveness of the proposed Baseline K-means algorithm in a distributed environment, where the coordinator and workers jointly process and learn from the evolving network traffic data. To execute the simulation, we first initialize an empty results dataframe to store the performance metrics (precision, recall, and F1-score) for each epoch. The simulation loop continues until less than 1000 elements remain in both the normalized data and the ground-truth labels. Within each epoch, the coordinator and workers process their respective data windows, and the performance metrics are computed and stored in the results dataframe.

**Figure 5:**
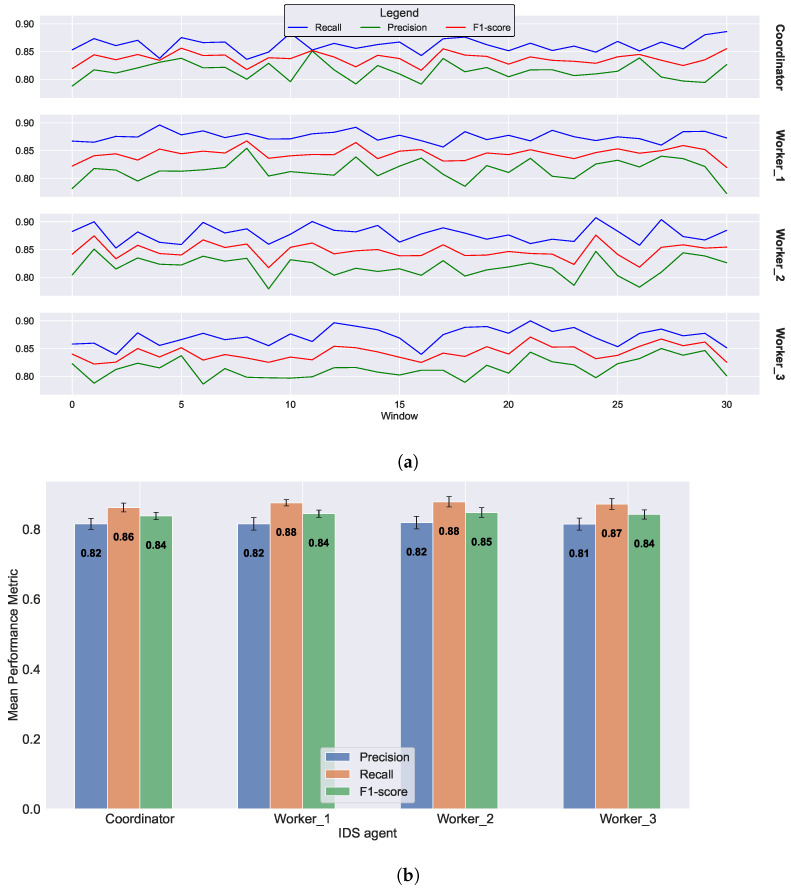
Federated IDS without merging operations. (**a**) Evolution of performance metrics for the coordinator and three workers over 30 epochs, each comprising a window of 1000 data points. (**b**) Average performance metrics for the coordinator and three workers after 30 epochs.

In our simulation, three scenarios were considered. The first scenario involves no merging operation; the coordinator conducts its initial training and exports its statistics. Each worker uses the initial statistics throughout all epochs or windows. In the second and third scenarios, there are 3 and 4 merge operations, respectively, occurring at specific points during the simulation. In these scenarios, the coordinator and workers share and update their respective statistics. The objective is to examine and determine the extent to which this merging step contributes to the overall system’s ability to adapt to the evolving nature of network traffic data. The results of the first scenario (no merging operation) are illustrated in Figure 5. As evident from the figure, the performance of the workers is comparable to, and occasionally slightly better than, that of the coordinator. The coordinator’s recall is approximately 0.86, while the workers have an average recall of 0.87. The average F1-score for all agents is around 0.84. The performance metrics exhibit minor variations in each window, depending on the processed data but remain close to the average values depicted in the bar plot (Figure 5b). No noticeable increasing or decreasing trend is observed in Figure 5a, indicating that the performance relies on the initial training and the baseline data learned by the coordinator. The exported statistics at the beginning remain constant, resulting in a similar performance for each data window.

**Figure 6:**
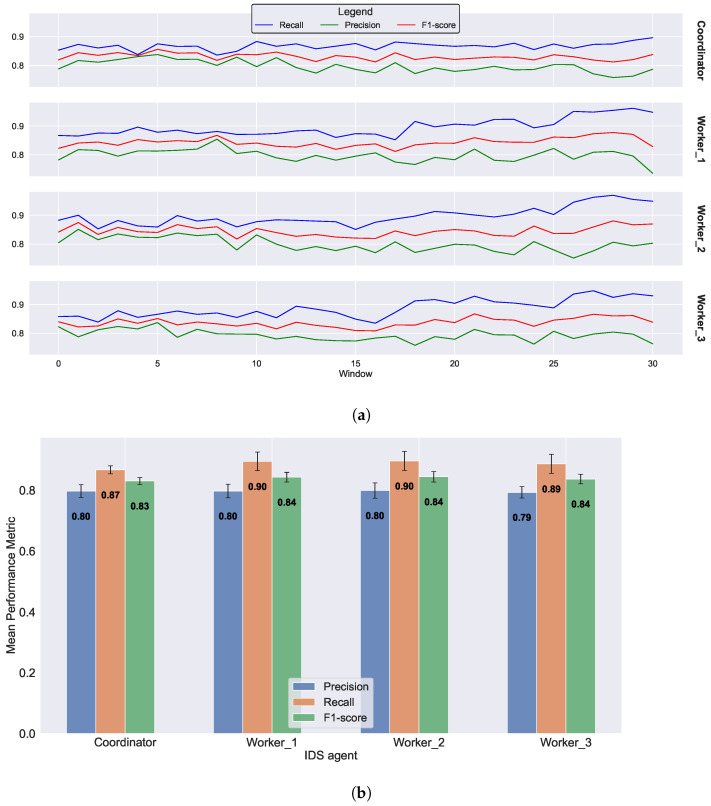
Federated IDS with three merging operations. (**a**) Evolution of performance metrics for the coordinator and three workers over 30 epochs, each comprising a window of 1000 data points. (**b**) Average performance metrics for the coordinator and three workers after 30 epochs.

The results of the second scenario are presented in Figure 6, where three merging operations occur at epochs 7, 14, and 21. From the figure, it is evident that the workers’ recall increases after each epoch involving a merging operation. The workers begin with an average recall of 0.87 at epoch 1 and conclude with an average recall of 0.94 at epoch 30 (Figure 6a). A comparison of the bar plots in Figure 6b reveals an increase in recall values in contrast to the scenario without merging operations. This observation supports our proposed approach, suggesting that the federated IDS can enhance the workers’ performance over time. Although the recall exhibits an increasing trend, a slight decrease in precision can also be observed. This phenomenon can be attributed to our approach’s prioritization of the true-positive rate over the false-positive rate. After each merging operation, the workers become more stringent in terms of rejecting distances greater than the dynamically evolving threshold. This measure is implemented to minimize the number of anomalous data points detected.

**Figure 7:**
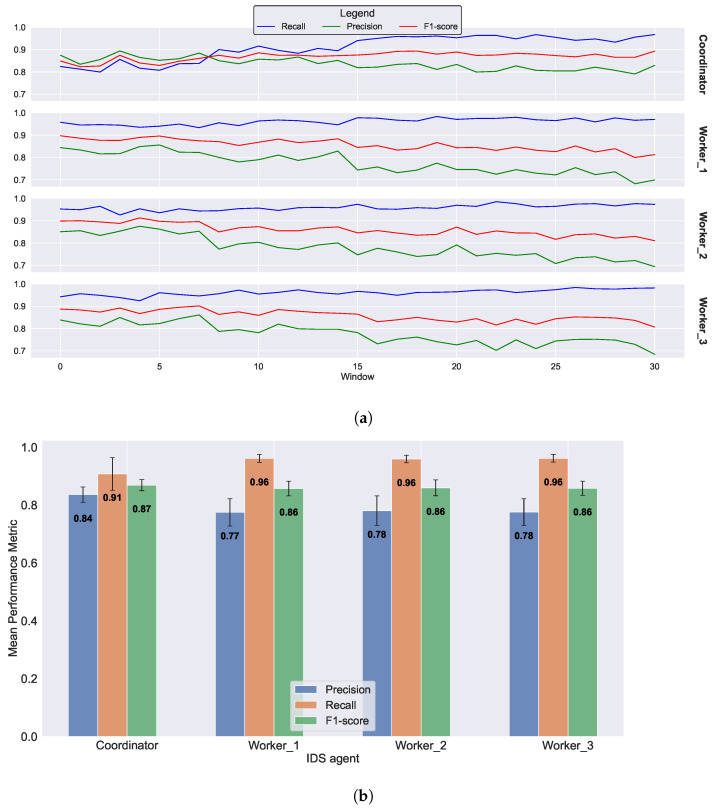
Federated IDS with four merging operations. (**a**) Evolution of performance metrics for the coordinator and three workers over 30 epochs, each comprising a window of 1000 data points. (**b**) Average performance metrics for the coordinator and three workers after 30 epochs.

The improvement in the true-positive rates over time is more evident in the results of the third scenario, as depicted in Figure 7. After 30 epochs, the workers achieve a recall of 0.97, as demonstrated in Figure 7a. Figure 7b reveals that the average recall for all workers is approximately 0.96, and the F1-score increases to 0.86. However, similar to the second scenario, the precision decreases to 0.78. Consequently, it is essential to consider the trade-off between precision and recall and to determine the appropriate intervals for merging operations. This consideration is crucial because, after each merging event, the worker IDS further prioritizes the true-positive rate.

### 4.4. Cross-Layer Federated Learning

This section discusses the simulation of cross-layer IDS where intrusion detection is applied after the cluster-based sampling proposed in our previous work [3], and shows the effect of the sampling on the IDS performance metrics. Next, this section highlights the importance of the sampling operation when implementing the IDS on a microcontroller in terms of processing time.

#### 4.4.1. IDS Performance Metrics

In this section, we explore the concept of cross-layer federated IDS, where a cluster-based sampling technique is applied before intrusion detection. We consider two scenarios involving a coordinator and a worker, each with three sampling rates: 0.75, 0.5, and 0.25. In the first scenario, the coordinator trains on baseline data, exports its statistics to the worker, and then the worker utilizes the same statistics for 11 epochs, processing 5000 data points per epoch. In the second scenario, two merging operations are added at epochs 3 and 7.

**Figure 8:**
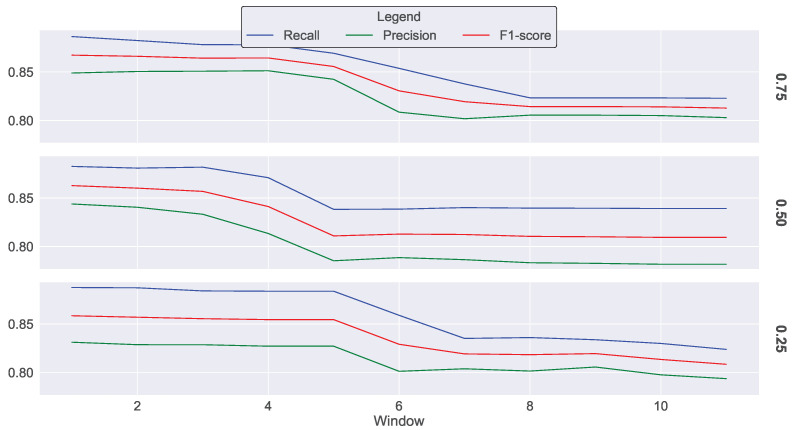
IDS performance metrics when applying cluster-based sampling with sampling rates of 0.75, 0.5, and 0.25, a change ratio of 0.5, and without merging operations.

Figure 8 illustrates the results of the first scenario, revealing that the performance of the IDS decreases with time when reducing the amount of data through cluster-based sampling. For example, at all three sampling rates, the worker starts with a recall value of 0.88 at Epoch 1 and concludes with a recall of 0.82 at Epoch 11. The performance drop is faster when the sampling rate is lowered, as depicted in Figure 8.

**Figure 9:**
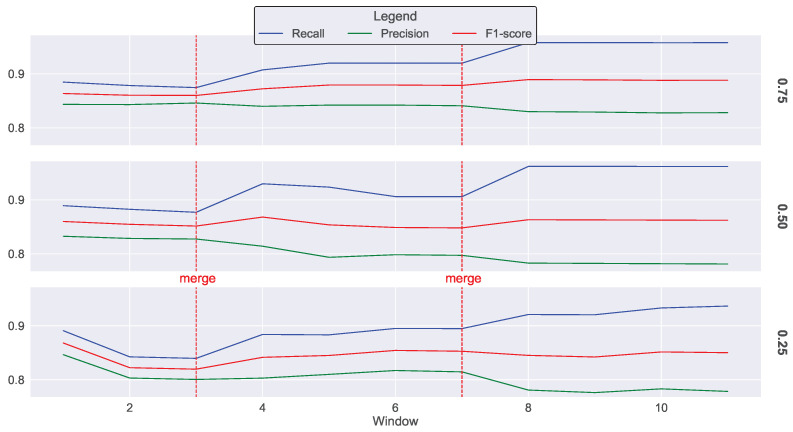
IDS performance metrics when applying cluster-based sampling with sampling rates of 0.75, 0.5, and 0.25, a change ratio of 0.5, and two merging operations.

Figure 9 showcases the significance of the proposed federated IDS in the second scenario. For sampling rates of 0.75 and 0.5, the recall decreases from 0.88 to 0.87 at epoch 3, and the sampling rate of 0.25 drops to 0.83. After the first merging operation, where the worker exports its statistics to the coordinator and receives the aggregated statistics, the recall increases to 0.90 for sampling rates 0.75 and 0.5 and to 0.89 for the sampling rate 0.25 by epoch 7. At epoch 11, the recall reaches 0.95 for sampling rates 0.75 and 0.5 and 0.93 for the sampling rate 0.25.

It should be noted that our simulation results may vary between runs due to the shuffling of NSL-KDD data, leading the worker to process different data windows in each simulation. However, in all runs, the worker consistently demonstrates a higher recall and F1-score at the final epoch compared to the first epoch. These findings validate the effectiveness of our proposed federated IDS and its ability to improve the performance of the workers over time through cooperation with the coordinator and the merging of statistics.

#### 4.4.2. Processing and Implementation

This section illustrates the implementation of the lightweight IDS on microcontrollers, specifically the Arduino Nano 33, to demonstrate its feasibility and efficiency in real-world applications. The IDS employed in this paper uses the Mahalanobis distance, which is a lightweight and easily implementable algorithm suitable for low-power devices such as microcontrollers. The coordinator exports the threshold, baseline mean, baseline covariance inverse, anomalous mean, and anomalous covariance inverse values to each worker. In the current implementation, these values were defined manually. In a real-world application, these values could be downloaded by the workers at the beginning of the process and after each merging operation. The C++ implementation of the function that calculates the Mahalanobis distance is computationally efficient and can be easily executed on resource-constrained devices like the Arduino Nano 33. This makes the entire federated IDS implementation suitable for low-power devices and applicable to a wide range of IoT systems.

**Figure 10:**
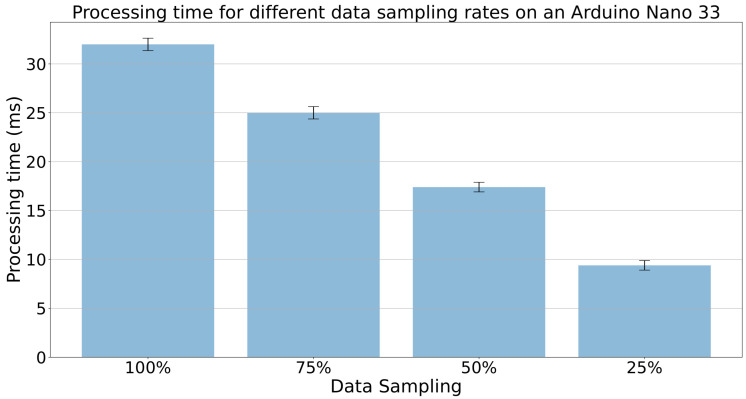
Processing time vs. sampling rate on an Arduino Nano 33.

To further improve the efficiency of the IDS, we simulated cluster-based sampling at various rates (0.75, 0.5, and 0.25) in addition to processing the whole dataset. Figure 10 demonstrates the benefits of using cluster-based sampling. As the sampling rate decreases, the amount of data stored in memory and the execution time of the IDS are reduced. This allows for faster processing times and increased efficiency, which is particularly important in resource-constrained environments. Figure 10 shows that the processing time in milliseconds reduces from around 32 milliseconds for a window of 100 data points, each containing six features, to 17 milliseconds when applying a sampling rate of 0.5, arriving at around 9 milliseconds with a sampling rate of 0.25.

## 5. Discussion

### 5.1. Experimental Results

In this section, we address three key observations derived from the experiments and their corresponding results. First, we aim to clarify the discrepancy in performance enhancement between the coordinator and the workers, as illustrated in Figure 6 and Figure 7. Second, we discuss the observed decline in precision, or the increase in the false-positive rate, when the coordinator and workers engage in a merging operation. Finally, we examine the third observation, which pertains to the cross-layer federated IDS simulation and, specifically, the performance degradation over time when utilizing cluster-based sampling.

Regarding the first observation, the primary reason the coordinator’s performance does not improve over time, as it does for the workers, is attributable to the workers not updating the coordinator with their inverse covariance matrix. It is important to emphasize that sharing the covariance matrix can be computationally demanding, particularly when the number of features is high. Computing the inverse covariance matrix on devices such as the Arduino Nano 33 or ESP32 can pose significant computational challenges, especially with a high number of features. Nevertheless, not transmitting the covariance matrix may result in a less accurate model, but this trade-off could be acceptable if the objective is to minimize computational and communication overheads. Utilizing the coordinator’s covariance matrix to calculate distances and define a threshold represents a viable alternative to transmitting the covariance matrix from the workers. This approach offers the benefit of reducing communication overheads since only the means and the distances need to be exchanged between the workers and the coordinator. By employing the coordinator’s covariance matrix, consistency in the threshold value can be maintained across all workers, even if their datasets differ. However, this method presupposes that the coordinator’s covariance matrix accurately represents the global dataset and that the data point distribution in each worker’s dataset closely aligns with the global distribution. If significant disparities exist in the data point distribution across workers, this approach may not yield results as accurate as those obtained using each worker’s local covariance matrix. In such cases, it may be better to use a hybrid approach where the workers send a compressed version of the covariance matrix, such as the diagonal elements or a subset of the matrix, which can be used to adjust the threshold value based on the local distribution of data points in each worker’s dataset. These cases could be studied in future works.

Upon examining the second observation in the experiments, it is evident that during the merging operation, where the worker exports its statistics to the coordinator and subsequently receives aggregated statistics, the recall of the worker increases, while its precision decreases over time. This phenomenon can be attributed to the IDS becoming stricter in terms of rejecting more packets or data points than before. In the provided implementation, the coordinator’s merge function computes the new means for baseline and anomalous data by averaging the coordinator’s and worker’s respective means. Additionally, it recalculates the threshold based on the percentile of distances between baseline data points and the updated baseline mean. As the merging operation advances, the threshold calculated by the coordinator typically becomes more conservative, resulting in the rejection of an increased number of data points. This happens because the recalculated threshold takes into account the specified percentile, as well as the minimum distance of a worker’s anomalous data points to the baseline mean. Consequently, all distances exceeding this minimum distance are regarded as anomalous. The worker’s merge function then updates its baseline and anomalous means, as well as the inverse covariance matrices and the threshold, based on the coordinator’s aggregated statistics. Consequently, this may increase false positives, therefore causing the precision to decrease. Meanwhile, the updated means and inverse covariance matrices help the IDS in detecting more true positives, increasing the recall. In future work, we intend to tackle this point and how the merging operation is defined and implemented to reduce the decrease in precision with time.

Concerning the third observation, which underscores a decreasing trend in the performance metrics of the IDS when the sampling rate is reduced over time, this phenomenon can be attributed to two primary factors. The first factor relates to the possibility that the initial baseline data may not accurately represent normal traffic. Since the dataset is shuffled for each run, the coordinator’s baseline data varies in each simulation. This variation might also mirror real-world scenarios in which the coordinator’s data may not precisely represent the pattern of data arriving at the worker. The second factor stems from the nature of the cluster-based sampling algorithm proposed in our previous work. This technique resembles a queue where each IoT device stores the sampled data in its memory, and new items are processed sequentially. Each new item is added to one of the clusters based on the Euclidean distance between the item and the center of each cluster. After assigning the item to a cluster, a decision is made on whether to include it in the corresponding cluster sample. This is determined by comparing the sampling error of the cluster sample with and without the item. If excluding the item leads to a higher sampling error, the new item will be sampled with a certain probability. Conversely, if including the item results in a higher sampling error, the item will be rejected and not added to the sample. To maintain a fixed cluster sample size, an item must be removed from the sample when the featured cluster size exceeds a change ratio constant. In this case, the item with the highest-ranked value in the current sample will be deleted. The decrease in IDS performance can be explained by the manner in which the cluster-based sampling technique selects data points for inclusion in the cluster sample. If the chosen data point is at the periphery of the benign distribution (i.e., the distance is not too close to the center and slightly exceeds the threshold), the IDS may regard it as a doubtful data point due to its conservative nature and subsequently reject it as an anomaly. However, this occasional decrease in performance can be mitigated through the merging operation, which enhances each worker’s performance.

### 5.2. Differential Analysis with Existing Techniques

This section reviews recent federated learning strategies for intrusion-detection systems (IDS) and compares them with the approach proposed in this paper. The authors in [54] employ an architecture very similar to the one we propose, concluding that utilizing an initially pre-trained model leads to better results. This mirrors the approach we adopt, initiating with a Baseline K-means model that has been pre-trained. Contrary to our work, which formulates the problem as a semi-supervised novelty detection task, they use a Deep Neural Network (DNN) and Deep Belief Network (DBN) for supervised multi-class classification. The work in [54] might be more suitable for IoT devices with substantial memory and computational capabilities, such as the Raspberry Pi 4, whereas our approach, due to the simplicity of the K-means algorithm compared to a neural network, can be embedded on more constrained devices like the ESP32.

The authors in [55] propose the Decentralized Online Federated Learning Intrusion Detection (DOF-ID), a collaborative learning system. Similar to the Baseline K-means, DOF-ID allows each IDS used for a cybersystem to learn from experiences gathered in other cybersystems, in addition to its own local data, without violating the data privacy of other systems. As in our paper, they start with the assumption that having labeled data is not feasible, so they apply a novelty detection strategy by training an auto-associative G-Network on data containing local benign network traffic. Unlike our work, their approach is decentralized; each node *n* communicates directly with other nodes to send locally learned IDS statistics and to receive those learned by others. The work in [55] focuses on decentralizing collaborative learning and optimizing IoT traffic processing, whereas our research emphasizes cross-layer anomaly detection. Our main objectives are to detect anomalies while reducing energy consumption by limiting the amount of data transmitted, using a cluster-based sampling strategy.

The authors in [31] propose an ensemble federated learning IDS at the gateway level. Similar to our work, they download an initial model trained on the server with a certain number of attacks to each gateway, enhancing it based on the local data of the IoT devices. Their ensemble approach efficiently addresses the problem of false positives. However, they employ a supervised approach for intrusion detection, which differs from the strategy of our paper, as we assume only a small number of benign data are available initially.

Each existing federated learning strategy has its own merits and underlying assumptions. For future work, we intend to incorporate various aspects of these works into our solution, such as decentralization and ensemble-based approaches.

## 6. Conclusions

This study proposes and analyzes a lightweight intrusion-detection system (IDS) for Internet of Things (IoT) devices, focusing on its implementation in microcontrollers and its applicability in real-world settings. Experiments and simulations have demonstrated the effectiveness and efficiency of the proposed IDS when implemented in a cross-layer federated learning framework, where a sampling operation precedes intrusion detection and the impact of cluster-based sampling on its performance.

The primary findings of this study highlight the influence of merging operations on the performance of the coordinator and workers, the trade-offs between precision and recall when sharing statistics between workers and the coordinator, and the factors affecting the performance trends when adjusting the sampling rate. These results provide valuable insights into the aspects that may influence the performance of the proposed IDS in existing IoT systems. Experiments and simulations indicate the suitability of the proposed lightweight IDS for IoT applications and the importance of using federated IDS to preserve privacy, offering a potential alternative for intrusion detection in resource-constrained contexts.

## Data Availability

The simulation code is publicly shared at [51].
